# HPLC Separation of All Aldopentoses and Aldohexoses on an Anion-Exchange Stationary Phase Prepared from Polystyrene-Based Copolymer and Diamine: The Effect of NaOH Eluent Concentration

**DOI:** 10.3390/molecules16075905

**Published:** 2011-07-14

**Authors:** Kadumi Inoue, Kei-ichi Kitahara, Yoshihiro Aikawa, Sadao Arai, Takako Masuda-Hanada

**Affiliations:** 1 Department of Human Environmental Sciences, Ochanomizu University, 2-1-1 Otuka, Bunkyo-ku, Tokyo 112-8610, Japan; 2 Department of Chemistry, Tokyo Medical University, 6-1-1 Shinjuku, Shinjuku-ku, Tokyo 160-8402, Japan

**Keywords:** carbohydrate, aldose, aldopentose, aldohexose, high-performance anion-exchange chromatography

## Abstract

To investigate the separations of all aldopentoses (ribose, arabinose, xylose and lyxose) and aldohexoses (glucose, galactose, allose, altrose, mannose, gulose, idose and talose) on the D_6_ stationary phase prepared by the reaction of chloromethylated styrene-divinylbenzene copolymer and *N*,*N*,*N*’,*N*’-tetramethyl-1,6-diaminohexane, we examined the effect of varying the concentration of the NaOH eluent on the elution orders. Separations of these aldoses were achieved using a 20 mM NaOH eluent. The elution behaviors of the aldoses were probably due to not only the individual p*K*_a_ values, but also the chemical structures of the cyclic aldoses.

## 1. Introduction

Carbohydrates are widely distributed in Nature, and are prime substances in many biological processes [1,2,3,4]. They are also used in the pharmaceutical and food industries [5,6]. Therefore, the effective separation and detection of carbohydrates is an important subject of investigation. However, the analysis of carbohydrates is difficult due to their structural diversities and the lack of chromophores. The hydroxyl groups of carbohydrates are partially ionized under highly alkaline conditions to form oxyanions, and thus carbohydrates can be separated by anion-exchange mechanisms. Currently, high performance anion-exchange chromatography (HPAE) at high-pH with electrochemical detection (ED) has been introduced as a highly sensitive and selective detection method for carbohydrates without the need for prior derivatization [7,8,9,10,11,12,13,14,15]. In this method, a limited number of sorbents has been reported: the electrostatically latex-coated pellicular polymeric-based anion-exchange sorbents [11] and the macroporous poly(styrene-divinylbenzene) sorbents with a trimethylammonium group [16,17].

**Figure 1:**
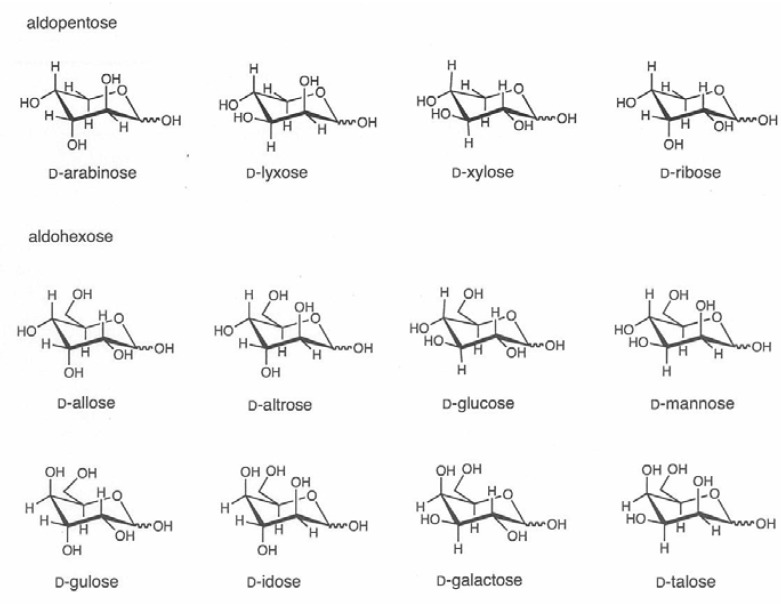
Chair forms of D-aldopentopyranoses and D-aldohexopyranoses studied.

We have previously reported the preparation of the novel D*_n_* anion-exchange stationary phases with both a quaternary nitrogen atom and a tertiary nitrogen atom by the reaction of porous particles of the chloromethylated styrene-divinylbenzene copolymer with *N*,*N*,*N*’,*N*’-tetramethyl-α,ω-diaminoalkanes (diamines) [18,19,20]. The HPAE-ED analyses of monosaccharides, disaccharides, and oligosaccharides using the 100 mM NaOH eluent were successfully performed. These results prompted us to investigate the separation of structurally very similar monsaccharides. In this paper, we will report the HPLC separation of all D-aldopentoses (D-ribose, D-arabinose, D-xylose and D-lyxose) and D-aldohexoses (D-allose, D-altrose, D-glucose, D-mannose, D-gulose, D-idose, D-galactose and D-talose) by an anion-exchange stationary phase prepared from polystyrene-based resin and a diamine. The chemical structures of all the aldoses are shown in Figure 1 as the chair forms of the D-aldopentopyranoses and D-aldohexopyranoses.

## 2. Results and Discussion

**Scheme 1:**
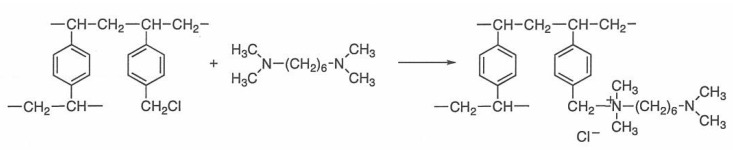
Synthesis of the anion-exchange stationary phase D_6_.

**Figure 2:**
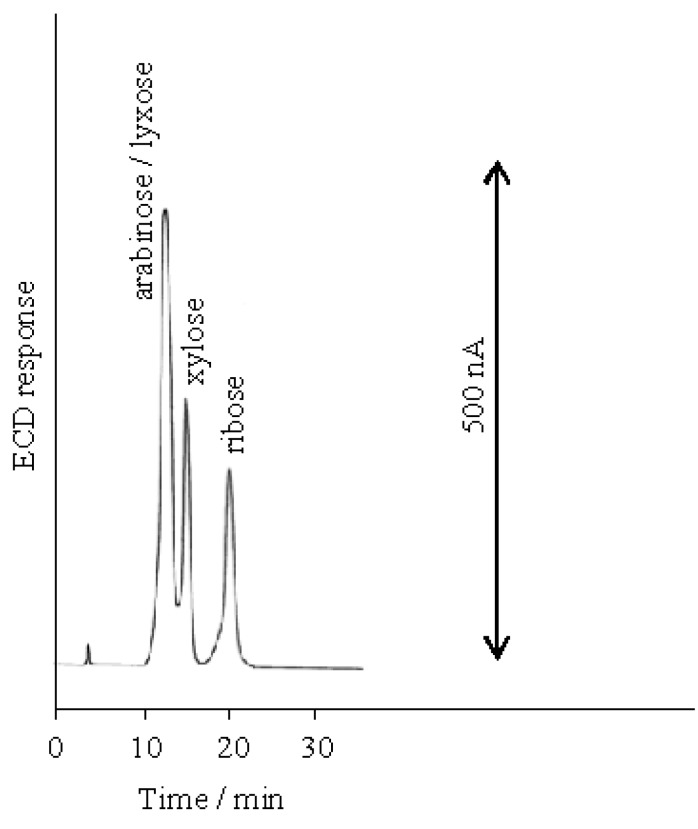
Separation of aldopentoses on the D_6_ stationary phase at 100 mM NaOH eluent.

Under alkaline conditions, the hydroxy groups of the carbohydrates are at least partially ionized. There are subtle differences in the p*K*_a_ values of the anomeric hydroxy group in carbohydrates as shown in Table 1 [21], and the separations of the various monosaccharides are achieved by anion-exchange sorbents. The separations of all the aldopentoses (D-ribose, D-arabinose, D-xylose and D-lyxose), each of which had a known p*K*a value, have been investigated on the D_6_ stationary phase obtained by the reaction of chloromethylated styrene-divinylbenzene copolymer with *N,N,N’,N’*-tetramethyl-1,6-diaminohexane (Scheme 1). The result using 100 mM NaOH as the eluent is shown in Figure 2, which shows that ribose (p*K*_a_ 12.11) eluted after xylose (p*K*_a_ 12.15). However, D-arabinose (p*K*_a_ 12.34) and D-lyxose (p*K*_a_ 12.11) co-eluted as a single peak and all the aldopentoses could not be resolved under this condition.

**Table 1:** The percentage compositions of aldoses in aqueous solution at equilibrium [22].

Aldose	p*K*_a_ [21] (25 °C)	temperature (°C)	pyranose		furanose	
			α (%)	β (%)	α (%)	β (%)
ribose	12.11	31	21.5	58.5	6.5	13.5
arabinose	12.34	31	60	35.5	2.5	2
xylose	12.15	31	36.5	63	<1	<1
lyxose	12.11	31	70	28	1.5	0.5
allose		31	14	77.5	3.5	5
altrose		22	27	43	17	13
glucose	12.28	31	38	62	・・	0.14
mannose	12.08	44	64.9	34.2	0.6	0.3
gulose		22	16	81	・・	3
idose		31	38.5	36	11.5	14
galactose	12.35	31	30	64	2.5	3.5
talose	□	22	42	29	16	13

**Figure 3:**
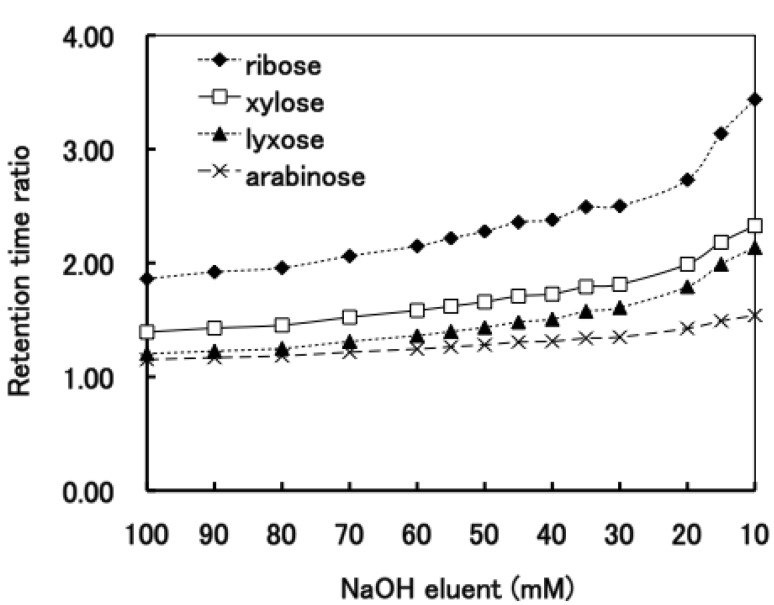
The elution behavior of aldopentoses on the D_6_ stationary phase over a range of NaOH concentrations.

**Figure 4:**
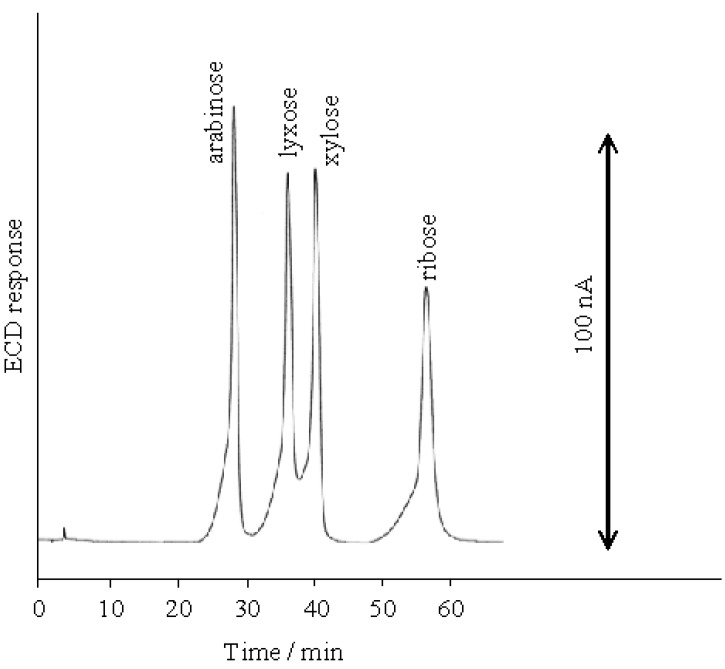
Separation of aldopentoses on the D_6_ stationary phase at 20 mM NaOH eluent.

The prolongation of the retention time of aldoses, caused by decreasing of the NaOH concentrations of eluents, would lead the better resolution of these aldoses [20]. Therefore, we examined the effect of varying the concentration of the NaOH eluent on the elution positions. Figure 3 shows that the retention time ratios of all the aldopentoses gradually increased in an almost linear manner with the decreasing concentration of the NaOH eluent from 100 to 30 mM, and below 30 mM NaOH, the ratios steeply increased. Although the retention time ratio of D-lyxose is nearly identical to that of D-arabinose from 100 to 80 mM NaOH, D-arabinose and D-lyxose could be effectively resolved at low NaOH concentrations. The optimal resolution of all the aldopentoses was achieved using the 20 mM NaOH eluent as shown in Figure 4. However, the elution sequence of the aldopentoses was different from the order of the p*K*_a_ values. Over the range of the studied NaOH concentrations, the elution order of the aldopentoses was D-arabinose (p*K*_a_ 12.34), D-lyxose (p*K*_a_ 12.11), D-xylose (p*K*_a_ 12.15), D-ribose (p*K*_a_ 12.11).

**Figure 5:**
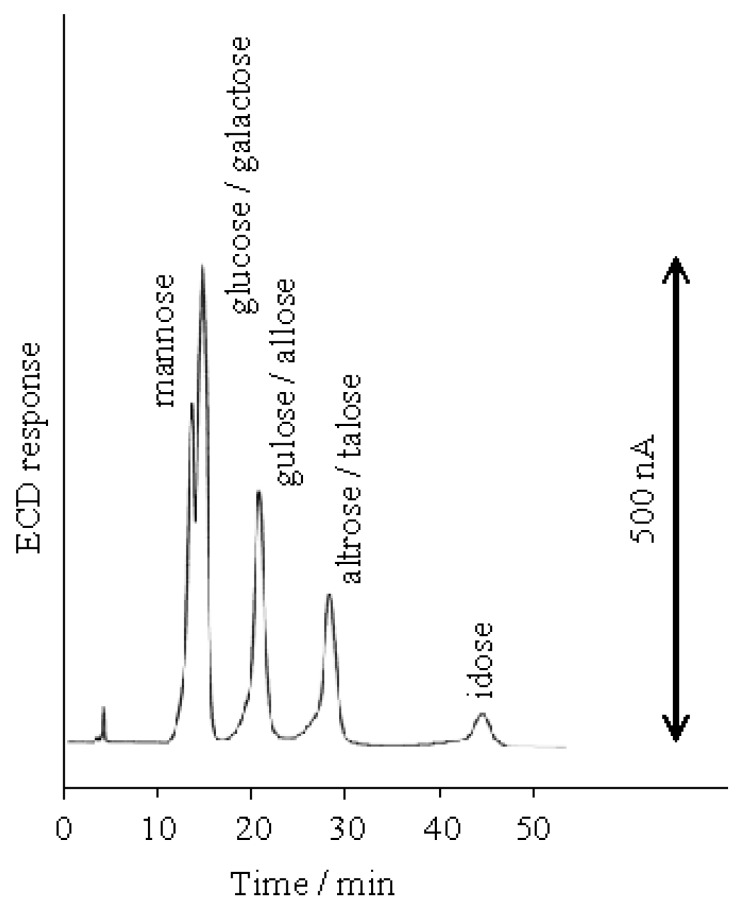
Separation of aldohexoses on the D_6_ stationary phase at 100 mM NaOH eluent.

**Figure 6:**
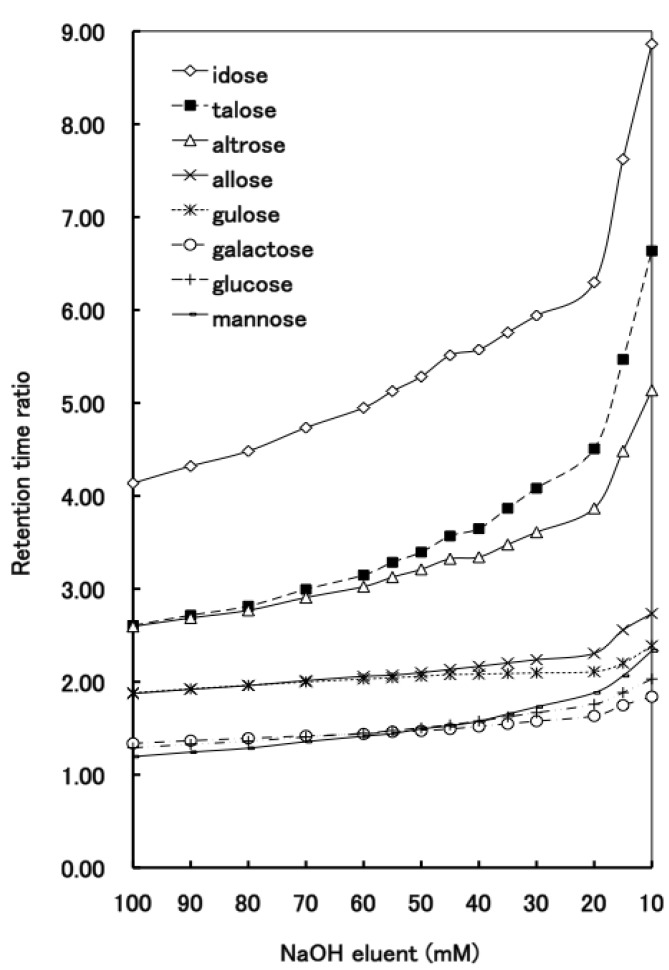
The elution behavior of aldohexoses on the D_6_ stationary phase over a range of NaOH concentrations.

**Figure 7:**
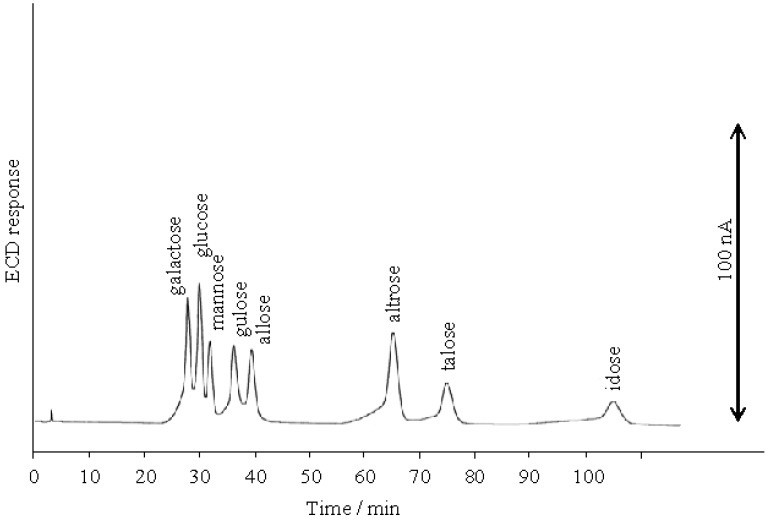
Separation of aldohexoses on the D_6_ stationary phase at 20 mM NaOH eluent.

The separation of all the aldohexoses (D-allose, D-altrose, D-glucose, D-mannose, D-gulose, D-idose, D-galactose and D-talose) was examined using 100 mM NaOH on the D_6_ stationary phase. Out of eight aldohexoses the p*K*a values of three aldohexoses were known (Table 1). Figure 5 shows that the aldohexoses were separated into two single peaks and three coupled peaks. In order to separate all the aldohexoses, we studied the effect of the NaOH eluent concentration. As shown in Figure 6, the elution behavior of the aldohexoses was almost similar to the tendency of the aldopentoses; the retention time ratios of all the aldohexoses gradually increased with the decreasing NaOH eluent concentration from 100 mM to 30 mM. Figure 3 and Figure 6 show that the retention time ratios of the aldoses steeply increased around 20 mM NaOH (pH 12.3) corresponding to the p*K*_a_ values of the aldoses. These results indicate that the dissociated aldoses strongly interact with the quaternary nitrogen atom of the stationary phase than the compepitive hydroxide ions in the eluent. The optimal resolution of all aldohexoses was achieved using the 20 mM NaOH elution on the D_6_ stationary phase as shown in Figure 7.

Although the change in the relative elution positions of the aldopentoses has not been observed as shown in Figure 3, it is noteworthy that the change in the elution orders of D-mannose, D-glucose and D-galactose was observed at concentrations between 60 mM and 30 mM. Using the 100 mM NaOH eluent, these aldohexoses were retained as follows: D-galactose = D-glucose > D-mannose. In contrast, at low NaOH concentrations (from 30 mM to 10 mM), these three aldohexoses were reasonably retained as follows: D-mannose (p*K*_a_ 12.08) > D-glucose (p*K*_a_ 12.28) > D-galactose (p*K*_a_ 12.35). Similar results have been reported by Mcguire [23].

It is well known that the anomeric hydroxy group of the pyranose form is more acidic than the other hydroxy groups [7]. However, the ionization of the hydroxy groups other than the anomeric one is possible. According to Rendleman’s review, the acidity of hydroxy groups in methyl D-glucopyranoside decreases in the order 2-OH >> 6-OH > 3-OH > 4-OH [24]. Koizumi also analyzed the positional isomers of the methyl ethers of D-glucose and concluded that the acidity of the monosaccharide is in the following order: 1-OH > 2-OH > 6-OH > 3-OH > 4-OH [25]. Since the individual hydroxy groups of the monosaccharides reveal the different p*K*a values, the ionization of the hydroxy groups other than anomeric one probably play important roles during elution.

Figure 3 and Figure 6 show that ribose (aldopentose) and idose, talose and altrose (aldohexose) were strongly retained overall compared with the other aldoses, especially at low NaOH concentrations (from 20 to 10 mM). These observations also suggested that besides the p*K*a values, additional factors for the elution characteristics of carbohydrates should be considered. The aldoses exist as an equilibrium between the pyranoses and furanoses; the percentage composition of the cyclic forms of monosaccharides is given in Table 1 [22]. Usually, in aqueous solution, aldopentoses and aldohexoses exist primarily in the six-membered pyranose form. However, Table 1 shows that out of twelve aldoses, four aldoses exist in the five-membered furanose form in a higher rate: D-ribose, 20%; D-altrose, 30%; D-talose, 29% and D-idose, 25.5%. It is noteworthy that aldoses possessing a higher percentage furanose composition are retained strongly at low NaOH concentrations. Lee [7] and Olechno *et al.* [26] suggested that strong binding ability of ribose and fructose with an anion exchange column may be due to their furanose form. These results suggest that the elution behaviors of the aldoses would probably correlate not only with the p*K*_a_ values, but also with the furanose forms.

## 3. Experimental

### 3.1. Materials

The porous particles of the chloromethylated styrene-divinylbenzene copolymer (diameter, 5 μm; pore size, 270 Å; divinylbenzene, 54%) were supplied by Nishio Industry (Tokyo, Japan). *N*,*N*,*N*’,*N*’-Tetramethyl-1,6-diaminohexane was from Tokyo Kasei (Tokyo, Japan).

D-Xylose, D-lyxose, D-mannose, D-allose, D-gulose, D-talose and 2-deoxygalactose used as the internal standards were purchased from Tokyo Kasei. D-Ribose, D-arabinose, D-galactose were purchased from Wako (Osaka, Japan). D-Glucose and D-idose were purchased from Sigma (St. Louis, MO, USA). D-Altrose was purchased from Acros (Geel, Belgium). Mixtures of the monosaccharides consisting of equimolar quantities were prepared as needed.

The D_6_ anion-exchange stationary phase was prepared by the reaction of the chloromethylated styrene-divinylbenzene copolymer and *N*,*N*,*N*’,*N*’-tetramethyl-1,6-diaminohexane according to our previous paper [18], as shown in Scheme 1 [found: C, 73.54; H, 8.54; N, 2.68%; the nitrogen content calculated for the diamine: 0.96 mmol/g (based on N)]. In our previous paper the nitrogen content of the anion-exchange stationary phase D_6_ was 0.96 mmol/g [18]. This result indicates the sufficient reproducibility of this reaction. 

The D_6_ stationary phase was suspended in 50 mL of water obtained from a Millipore Milli-Q system (Millipore Corp., Bedford, MA), sonicated for 5 min and packed into a 250 × 4.6 mm I.D. polyether ether ketone (PEEK) column using 100 mM NaOH as the mobile phase at a constant pressure of 200 kg cm^−2^ by a Shimadzu LC-10AD pump (Kyoto, Japan). The sodium hydroxide solutions were prepared by the dilution of a 50% (w/w) stock NaOH solution with Milli-Q water. All mobile phases were deaerated by dispersed helium. After each run, the column was eluted with 100 mM NaOH at 1.0 mL/min for 20 min for cleaning and reequilibrating to the starting conditions.

### 3.2. Equipment

The HPLC experiments were performed using a Shimadzu LC-10AD pump (Kyoto, Japan) with a CHRATEC VI-501PS electrochemical detector (Kyoto, Japan) consisting of an amperometric flow-through cell with a Ni-Ti alloy working electrode and a silver-silver chloride reference electrode [19,20,27,28,29,20,27]. The Ni-Ti alloy wires [Ni-Ti: NI205100 (55:45, w/w), 0.8 mm diameter] were purchased from Goodfellow (Cambridge, UK). The Ni-Ti working electrode was made by embedding the Ni-Ti wire into a teflon block fitted to a CHRATEC (Kyoto, Japan) flow cell. The optimal detection potential for the Ni-Ti electrode is 500 mV in the NaOH eluents. Samples were injected using a non-metal Rheodyne (Cotati, CA, USA) Model 9125 injection valve.

### 3.3. Chromatographic Conditions and Measurements

The NaOH solution (50% w/v) as a stock solution was used to adjust the requied mobile phase concentration. The analysis of the aldoses employed isocratic elution at the flow rate of 1.0 mL/min with NaOH eluents varying from 10 mM–100 mM.

Carbohydrate stock solutions were prepared by dissolving each aldose in Milli-Q water at a concentration of 0.1 wt%, and filtered through a 0.45 mm-membrane filter. Before injecting mixtures of the aldopentoses and aldohexoses, we identified each aldose by injecting 2 μL of the diluted stock solution of 0.01 wt% concentration one by one. For the separation of aldopentose mixture and aldohexose mixture, sample solutions were prepared by mixing of equal volume of the carbohydrate stock solutions, and 1–2 μL of these solutions were injected to HPLC. The elution position of each aldose was expressed as the ratio of the retention time of the internal standard, 2-deoxy-galactose, at each NaOH concentration [23]. 2-Deoxy-galactose was selected as an internal standard because of eluting before all of the aldopentoses and aldohexoses without co-eluting.

## 4. Conclusions

Using the D_6_ stationary phase prepared by the reaction of chloromethylated styrene-divinylbenzene copolymer and *N*,*N*,*N*’,*N*’-tetramethyl-1,6-diaminohexane, the HPAE-ED separations of all the aldopentoses and aldohexoses were efficiently performed with a 20 mM NaOH eluent. To the best of our knowledge, this is the first report on the resolutions of all the aldopentoses and aldohexoses. The elution behaviors of the monosaccharides are probably not only dependent on the individual p*K*a values, but also on their chemical structures.
